# *Lactobacillus* species do not produce 1-acetyl-β-carboline

**DOI:** 10.1038/s41467-024-50683-5

**Published:** 2024-08-01

**Authors:** Tomás Herraiz, Ana Sánchez-Arroyo, Blanca de las Rivas, Rosario Muñoz

**Affiliations:** Instituto de Ciencia y Tecnología de Alimentos y Nutrición (ICTAN-CSIC). Spanish National Research Council (CSIC), José Antonio Nováis 6, 28040, Madrid, Spain

**Keywords:** Small molecules, Bacteria

**arising from** J. MacAlpine et al. *Nature Communications* 10.1038/s41467-021-26390-w (2021)

In a recent article entitled “A small molecule produced by *Lactobacillus* species blocks *Candida albicans* filamentation by inhibiting DYRK1-family kinase”, MacAlpine et al.^1^ reported that a small molecule identified as 1-acetyl-β-carboline (1-ABC) was produced by lactobacilli and blocked the filamentation of the pathogenic yeast *Candida albicans*. MacAlpine et al. found that culture media of lactobacilli blocked *Candida albicans* filamentation, which is a virulence trait, and attributed this action to 1-ABC, proposing a mechanism of action due to the activity of this β-carboline as an inhibitor of DYRK1 kinase. Owing to our interest in β-carbolines and lactic acid bacteria, we have studied the production of 1-ABC by several lactobacilli, including two strains used by MacAlpine et al., and our results do not confirm that lactobacilli produce this β-carboline. Instead, 1-ABC is produced by a reaction of L-tryptophan in the media with methylglyoxal (MGO). Noticeably, a recent metabolomic study of a *Lactobacillus* strain^2^ did not find 1-ABC either.

*Candida albicans* pathogenesis is affected by the ability to switch between yeast and filamentous morphologies, and this transition is important for virulence^1^. Interactions of *C. albicans* with other members of the microbiota such as lactobacilli regulate virulence^2,3^. In particular, lactobacilli antagonize *C. albicans* proliferation, hypha formation, and biofilm formation, and can kill *C. albicans*^2,4,5^. However, the mechanisms by which lactobacilli prevent *C. albicans* overgrowth and superficial mucosa invasion remain unclear^6^. In their work, MacAlpine et al.^1^ reported that “a molecule secreted by *Lactobacillus* is sufficient to repress *C. albicans* morphogenesis in response to diverse filament-inducing cues in culture” and linked this activity to 1-ABC by using a bioassay-guided fractionation approach. Furthermore, they identified kinase Yak1 as the target of 1-ABC and other synthetized β-carbolines (e.g., 1-ethoxycarbonyl-β-carboline) and proved that β-carbolines were active against yeast filamentation by inhibition of Yak1 in several co-culture models and a rat catheter infection model.

We investigate β-carboline alkaloids from natural products along with their biotransformation and activity^7,8^ and the bioactive compounds produced by lactic acid bacteria, particularly *Lactiplantibacillus plantarum*^9,10^. We therefore found the results of MacAlpine et al.^1^ very exciting because they suggested a pathway to produce bioactive β-carboline alkaloids by microbiota having an impact on the human health. Therefore, we pursued the production of 1-ABC by lactobacilli. We assayed four common lactic acid bacterial strains, namely *Lactiplantibacillus plantarum* WCFS1*, Lacticaseibacillus rhamnosus* GG (ATCC 53103), and two strains assayed by McAlpine et al., *L. rhamnosus* R0011 and *Lactobacillus helveticus* R0052. The strains were grown in MRS media and the production of 1-ABC was analyzed by HPLC and HPLC-MS (Supplementary Fig. 1 and 2). We did not find significant amounts of 1-ABC produced by these strains in cultures up to 72 h when compared with MRS control media (Fig. 1a and Supplementary Figs. 1 and 2). In contrast, MacAlpine et al. reported 1-ABC production by lactobacilli in cultures at 18 h. We noticed that MRS media contain the amino acid tryptophan that mostly remained unconsumed by lactobacilli in the media (Fig. 1b). As β-carbolines arise from L-tryptophan^7,8^, we thought that this amino acid could be a precursor of 1-ABC. Therefore, we studied whether an eventual formation of 1-ABC could arise from L-tryptophan present in the MRS, and found out that the compound 1-ABC was produced in the media in the presence of added MGO (Fig. 1d and Supplementary Figs. 3 and 4). 1-ABC was produced in the MRS media by a reaction between MGO and L-tryptophan (Fig. 1c) along with the consumption of this amino acid (Fig. 1f). This experiment was repeated with MRS media adjusted to pH 4 (simulating the acidic pH of MRS media after lactobacilli growth) and the concentration of 1-ABC increased substantially (Fig. 1e and Supplementary Figs. 3 and 4) along with a higher removal of L-tryptophan (Fig. 1f).

**Fig. 1:**
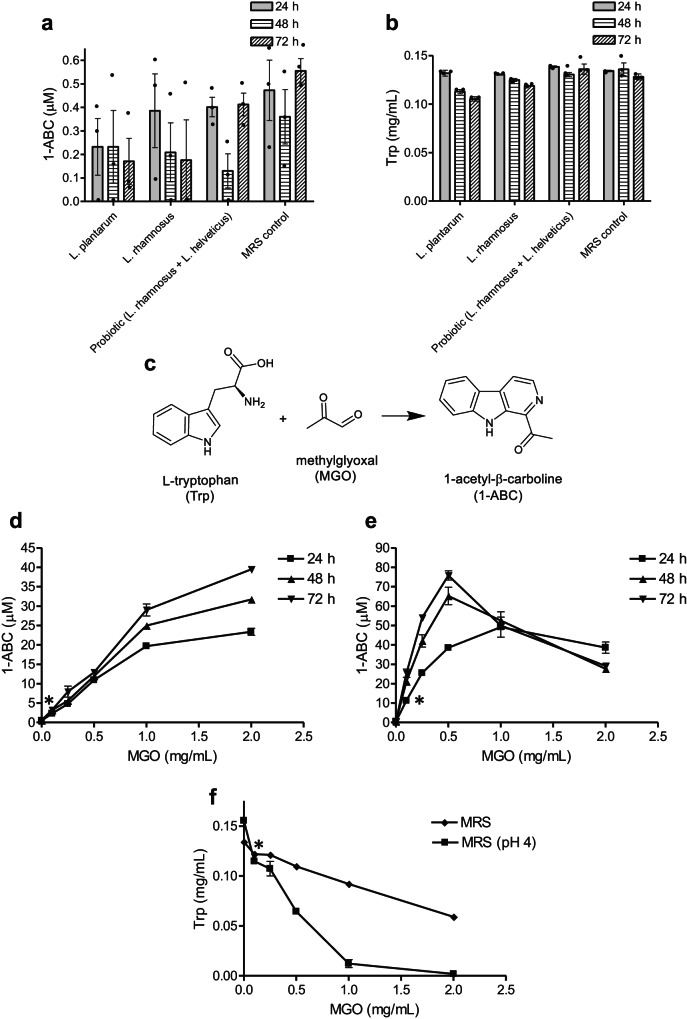
1-Acetyl-β-carboline (1-ABC) in control MRS media, *lactobacilli-*grown media, and MRS added with methylglyoxal (MGO). **a** Concentrations of 1-ABC in MRS control media and in MRS media grown with *L. plantarum* WCFS1, *L. rhamnosus* GG, and probiotic strains *L. rhamnosus* R0011 and *L. helveticus* R0052 incubated at 37 °C under anaerobic conditions for 24, 48 and 72 h. The MRS control media was incubated in the same conditions as the MRS media inoculated with lactobacilli. No significant differences (*p* > 0.05) were found among lactobacilli cultures and MRS control or among incubation time. **b** Content of L-tryptophan (Trp) in MRS and in the culture media of lactobacilli. The MRS control media was incubated in the same conditions as  the MRS media inoculated with lactobacilli. No significant differences were found between cultures or time. **c** Reaction of L-tryptophan with methylglyoxal (MGO) to afford 1-ABC. **d** Formation of 1-ABC in MRS media with added MGO and incubated at 37 °C in anaerobic conditions at 24, 48 and 72 h. Significant differences (*p* < 0.05) were found with the addition of MGO at 0.1 mg/mL and above vs MRS control. **e** Formation of 1-ABC in MRS media adjusted to pH 4 with added MGO and incubated in anaerobic conditions at 37 °C for 24, 48, and 72 h. Significant differences (p < 0.05) were found with the addition of MGO at 0.1 mg/mL and above vs MRS control. **f** Removal of L-Trp from MRS media with the addition of MGO (37 °C for 24 h). Significant differences (*p* < 0.05) were found with the addition of MGO at 0.1 mg/mL and above vs MRS control. Data represent mean ± SEM (*n* = 3). Significance (*) was determined using unpaired Student’s *t* test.

Our results indicate that 1-ABC was not produced by lactobacilli grown in MRS media. In contrast, 1-ABC was produced by a reaction between L-tryptophan present in the media and MGO. We used the same experimental conditions of MacAlpine et al.^1^, including MRS media, anaerobic growth conditions, incubation temperature (37 °C), and followed the culture for up to 3 days (compared with 18 h), and we used four different lactobacilli strains including two strains also assayed by MacAlpine et al.^1^. Nevertheless, some experimental details could have been different among both studies such as the volume of culture processed. Unfortunately, MacAlpine et al. did not give the concentrations of 1-ABC produced in their bacterial cultures nor the amount of 1-ABC isolated from their cultures for characterization purposes. Contrary to their findings, our results do not confirm that lactobacilli species produce significant amounts of this β-carboline as a metabolite. In recent work, Alonso-Roman et al.^2^ studied the interaction between the metabolism of *L. rhamnosus* ATCC 7469 and *C. albicans* WT SC5314 and these authors mentioned in the discussion that they did not find 1-ABC in their metabolomic study, and attributed this result to different culture conditions or a possible metabolism of the compound. However, we did not observe 1-ABC during the growth of lactobacilli, and therefore its metabolic removal is unlikely. Moreover, as 1-ABC arises from L-tryptophan that mostly remained unconsumed (Fig. 1b), its formation and further metabolism by lactobacilli can be ruled out.

As our results question that lactobacilli produce 1-ABC, another explanation for the presence of 1-ABC in the cultures of MacAlpine et al., might be offered. As seen here, this β-carboline arises from L-tryptophan present in the MRS media following a reaction with MGO. Then, one possibility is that MGO might have been somehow present in the media and reacted with tryptophan to give the β-carboline. MGO can be formed as a secondary product during glycolysis. Some microorganisms are able to produce MGO, and this metabolite has been the object of notable attention because it modulates survival and toxicity in bacteria^11^. Microorganisms containing the enzyme methylglyoxal synthase (MgsA) can produce MGO, and this enzyme could have led to the generation of MGO, and subsequently 1-ABC. However, the enzyme methylglyoxal synthase is rarely present in lactobacilli, including those species employed here and in the MacAlpine et al. study (NCBI Protein Database, https://www.ncbi.nlm.nih.gov/mesh/; Ghandi et al.^12^). Therefore, the production of MGO and 1-ABC by lactobacilli is unlikely. Another possibility is that a chemical source of MGO was incorporated into the cultures. MGO is produced as a degradation product of carbohydrates under heating^7^, and in fact, 1-ABC was already present in the MRS culture media, although in trace or minor amounts (Fig. 1a and Supplementary Figs. 1 and 2). Moreover, we observed that 1-ABC levels increased in the MRS media following a longer autoclave heating (Supplementary Fig. 5).

Finally, we must recognize and highlight that a main part of the work of MacAlpine et al.^1^ dealt with the inhibition of *C. albicans* filamentation, where they reported that synthetized 1-ABC and other β-carbolines blocked filamentation by the inhibition of DYRK1 kinase, and suggested that β-carbolines could be good scaffolds against *Candida* filamentation. Of course, those results are not the subject of this Matters Arising. In fact, β-carbolines are known as good kinase inhibitors^13^. However, our results do not confirm that lactobacilli produce 1-ABC, and therefore the inhibitory effects of lactobacilli on *C. albicans* filamentation could not be due to this β-carboline.

## Methods

### Bacterial strains and growth conditions of cell cultures

*Lactiplantibacillus plantarum* (formerly *Lactobacillus plantarum*) WCFS1 and *Lacticaseibacillus rhamnosus* (formerly *Lactobacillus rhamnosus*) GG strains used in this study were kindly provided by Dr. Michael Kleerebezem (NIZO Food Research, The Netherlands) and Dr. J. M. Landete (INIA, CSIC, Spain), respectively. The Lallemand´s property strains, *L. rhamnosus* R0011 (Rosell®−11) and *Lactobacillus helveticus* R0052 (Rosell®−52) were obtained from the commercial probiotic Lacidofil® (Lallemand). Lactobacilli strains were grown at least in triplicate in MRS broth (Pronadisa, Spain) (10 mL) at 37 °C in anaerobic jar. Aliquots were taken at 24, 48, and 72 h, centrifuged at 12,000 × *g*, and analyzed by HPLC and HPLC-MS.

### Production of 1-ABC from MGO in absence of lactobacilli

MRS sterilized culture media prepared for lactic acid bacteria growth (10 mL), and the same MRS media adjusted to pH 4 (10 mL), were added with MGO (Sigma) to reach from 0 to 2 mg/mL final concentrations, and incubated at 37 °C in anaerobic jar for 72 h. Aliquots were taken at 24, 48, and 72 h, and analyzed by HPLC and HPLC-MS. On the other hand, MRS culture media was subjected to longer autoclave sterilization (150 min vs 20 min) and analyzed by HPLC and HPLC-MS.

### Chromatographic analysis by HPLC and HPLC-MS

The chromatographic analysis of 1-ABC in cultures was performed with a 1050 high-performance liquid chromatograph (Agilent Technologies) coupled to 1100 series DAD. The separation was carried out with a 150 mm × 3.9 mm, 5 µm, Novapak C18 column (Waters). The eluents were 50 mM ammonium phosphate buffer adjusted to pH 3 with 85% phosphoric acid (Eluent A) and 20% of eluent A in acetonitrile (Eluent B). The gradient was set from 0% to 32%B in 8 min, then 90%B at 12 min. The flow rate was 1 mL/min, oven temperature was 40 °C, and the injection volume was 20 µL. The concentration of 1-ABC and L-tryptophan were obtained with calibration curves of synthetized 1-ABC standard and L-tryptophan and analyzed by HPLC with absorbance detected at 280 nm. Compound identification was accomplished by HPLC-MS (Electrospray ionization, ESI) with an HPLC-MS Waters Alliance e2695 Separations Module coupled to a Waters 2998 Photodiode Array Detector, and a quadrupole Waters Acquity QDa mass spectrometer working under positive electrospray ionization (ESI+) with a Capillary Voltage of 0.8 kV and Cone Voltages (10, 20, and 40 V). Chomatographic separation was accomplished with a 2.1 × 5 mm Waters Sunfire C18 VanGuard Cartridge (precolumn) and a 2.1 × 100 mm, 3 µm, 100 Å, C18 Atlantis T3 column (Waters). The chromatographic conditions were set from 15% to 90% acetonitrile containing 0.1% formic acid in 10 min, and then 100% acetonitrile at 12 min. The flow rate was 0.35 mL/min and the injection volume 1-3 µL. The mass spectra acquisition was from 85 to 1250 amu, and 1-ABC gave mainly the ion at *m/z* 211 (M + H)^+^, and in higher fragmentation (V = 40), the fragments at *m/z* 193 and 169.

### Reporting summary

Further information on research design is available in the Nature Portfolio Reporting Summary linked to this article.

### Supplementary information

Supplementary Information

Reporting Summary

### Source data

Source Data

## Data Availability

The data supporting the finding of the study are in the Article and supplementary files. Source data are provided with this paper.
