# Supplementary material for: Lactobacillus species do not produce 1-acetyl-β-carboline
Source: Nat Commun. 2024 Aug 1;15:6442. doi: 10.1038/s41467-024-50683-5 (PMC11294554; doi:10.1038/s41467-024-50683-5)
Supplement: Supplementary file 1 — Supplementary Information [file 41467_2024_50683_MOESM1_ESM.pdf]

Supplementary information

Lactobacillus species do not produce 1-acetyl-β-carboline

Tomás Herraiz*, Ana Sánchez-Arroyo, Blanca de las Rivas, Rosario Muñoz

Instituto de Ciencia y Tecnología de Alimentos (ICTAN-CSIC). Spanish National Research Council (CSIC). Jose Antonio Novais 6, Madrid, Spain

*Corresponding author: tomas.herraiz@csic.es

ARISING FROM from MacAlpine et al. Nature Communications https://doi.org/10.1038/s41467-021-26390-w (2021)

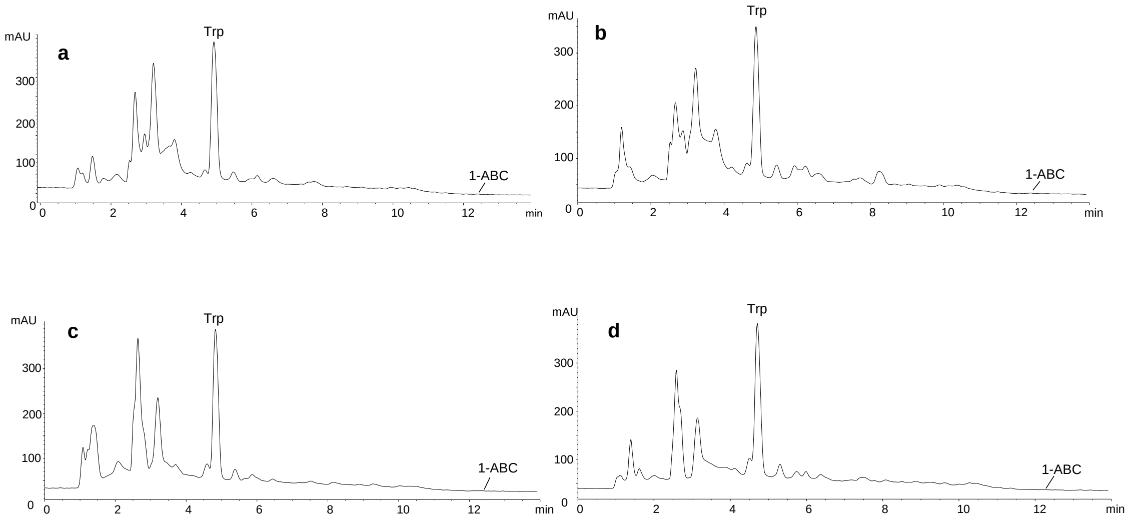

Supplementary Fig. 1. HPLC chromatograms (detection at 280 nm). **a** Control MRS. **b** MRS culture inoculated with *L. plantarum* WCFS1 (37ºC, 48 h)**. c** MRS culture with *L. rhamnosus* GG (37ºC, 48 h)*.* **d** MRS culture with probiotic strains *L. rhamnosus* R0011 and *Lactobacillus helveticus* R0052 (37ºC, 48 h). The MRS control media was incubated in the same conditions than the MRS media inoculated with lactobacilli. Trace amounts of 1-ABC (1-acetyl-β-carboline) are detected both in MRS control and lactobacilli cultures and confirmed by HPLC-MS. Trp: L-tryptophan.

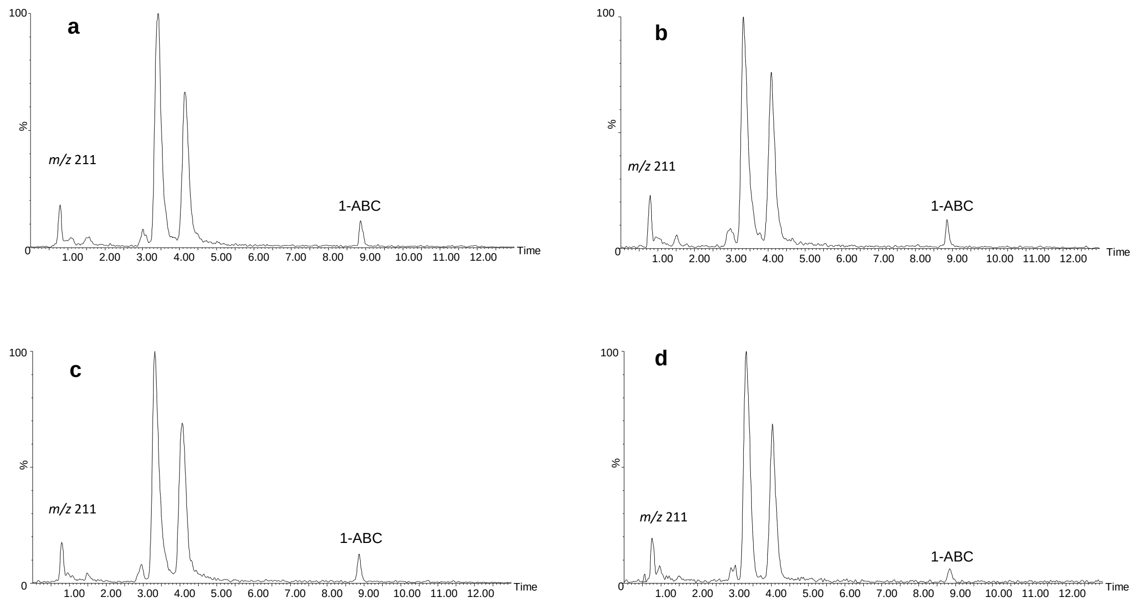

Supplementary Fig. 2. 1-ABC analysis by HPLC-MS (ESI+, *m/z* 211). **a** Control MRS. **b** MRS media inoculated with *L. plantarum* WCFS1 (37ºC, 48 h)**. c** MRS media inoculated with *L. rhamnosus* GG (37ºC, 48 h)*.* **d** MRS media inoculated with probiotic strains *L. rhamnosus* R0011 and *L. helveticus* R0052 (37ºC, 48 h). 1-ABC was present in both in MRS control and lactobacilli cultures.

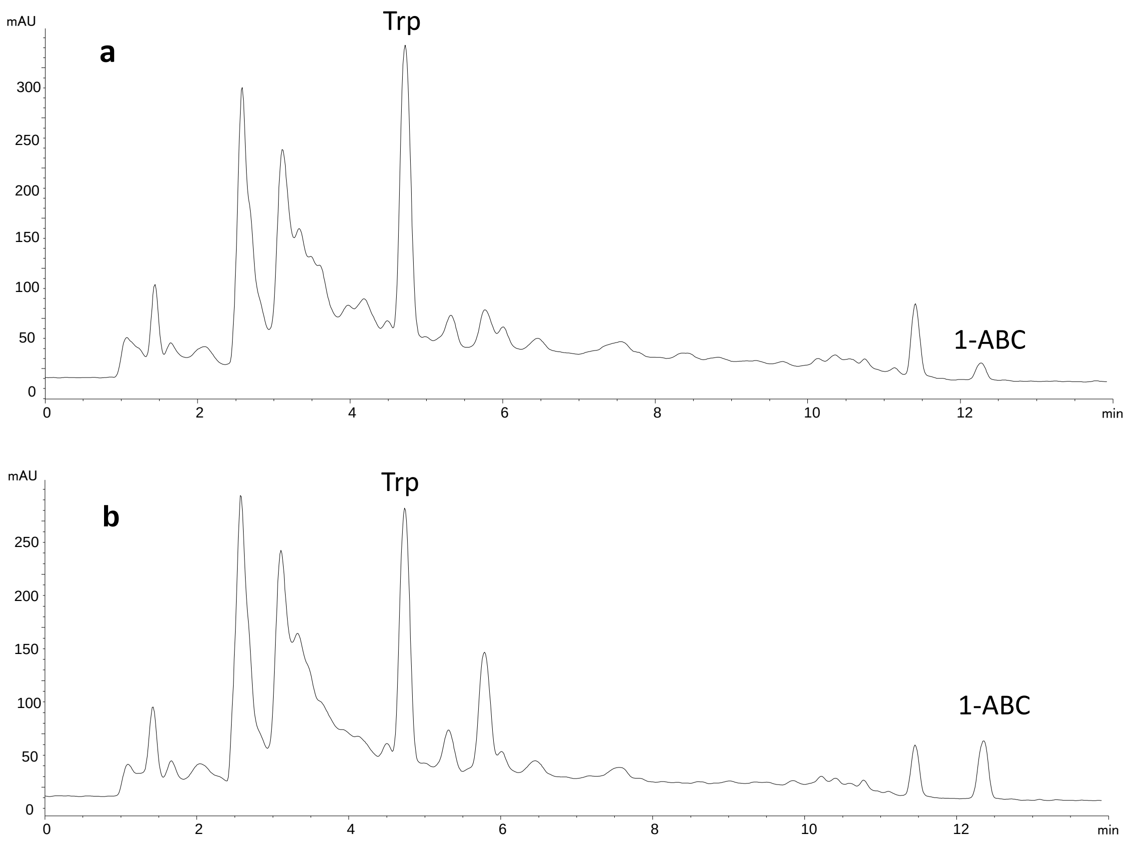

Supplementary Fig. 3. HPLC chromatograms (detection at 280 nm). **a** MRS added with 0.5 mg/mL methylglyoxal (MGO) (37ºC, 48 h). **b** MRS adjusted to pH 4 and added with 0.5 mg/mL MGO (37 ºC, 48 h). 1-ABC was produced in the reaction of L-Trp with MGO.

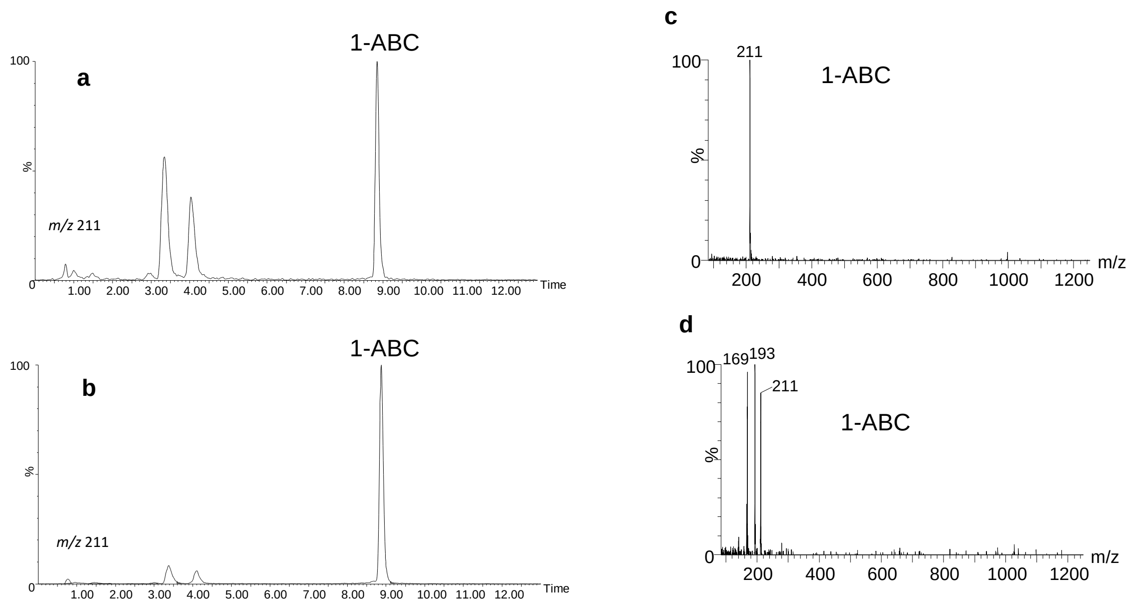

Supplementary Fig. 4. HPLC-MS chromatograms (ESI+, *m/z* 211). **a** MRS added with 0.5 mg/mL MGO (37ºC, 48 h). **b** MRS adjusted to pH 4 and added with 0.5 mg/mL MGO (37 ºC, 48 h). **c** Mass spectrum of the 1-ABC produced in the reaction of L-trp with MGO in low fragmentation voltage (V=10) with *m/z* 211 $(M+H)^+$ **d** Mass spectrum of 1-ABC in higher fragmetation (40 V) with ions at *m/z* 211 $(M+H)^+$ and fragments at *m/z* 193 and 169.

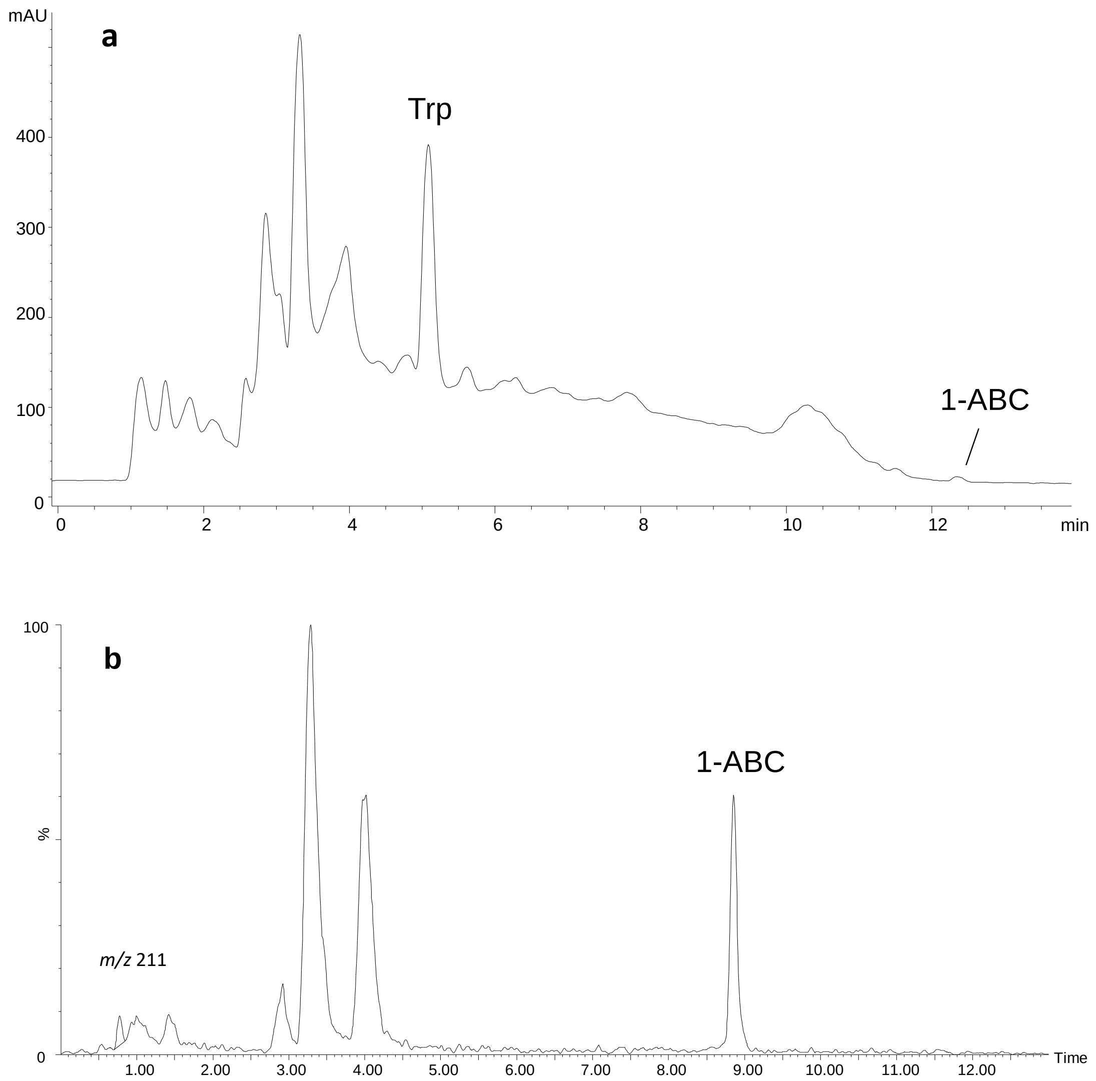

Supplementary Fig 5. Formation of 1-ABC in MRS after longer autoclave heating (150 min). a HPLC chromatogram (280 nm)**. b** HPLC-MS chromatogram (*m/z* 211). Concentration of 1-ABC increased from 0.5 µM in MRS control to 4.3 µM.
